# Implementation of Smartphone Systems to Improve Quality of Life for People With Substance Use Disorder: Interim Report on a Randomized Controlled Trial

**DOI:** 10.2196/35125

**Published:** 2022-07-14

**Authors:** David Gustafson Sr, Julie Horst, Deanne Boss, Kathryn Fleddermann, Nora Jacobson, Mathew Roosa, J Charles Ross, Rachel Gicquelais, Olivia Vjorn, Tracy Siegler, Todd Molfenter

**Affiliations:** 1 Department of Industrial and Systems Engineering University of Wisconsin-Madison Madison, WI United States; 2 Institute for Clinical and Translational Research University of Wisconsin-Madison Madison, WI United States; 3 School of Nursing University of Wisconsin-Madison Madison, WI United States

**Keywords:** mobile technology, coaching, substance use disorder (SUD) treatment, technology implementation model, NIATx

## Abstract

**Background:**

Researchers have conducted numerous studies seeking to understand how to improve the implementation of changes in health care organizations, but less focus has been given to applying lessons already learned from implementation science. Finding innovative ways to apply these findings efficiently and consistently will improve current research on implementation strategies and allow organizations utilizing these techniques to make changes more effectively.

**Objective:**

This research aims to compare a practical implementation approach that uses principles from prior implementation studies to more traditional ways of implementing change.

**Methods:**

A total of 43 addiction treatment sites in Iowa were randomly assigned to 2 different implementation strategies in a randomized comparative effectiveness trial studying the implementation of an eHealth substance use disorder treatment technology. One strategy used an adaptation of the Network for the Improvement of Addiction Treatment (NIATx) improvement approach, while the other used a traditional product training model. This paper discusses lessons learned about implementation.

**Results:**

This midterm report indicates that use of the NIATx approach appears to be leading to improved outcomes on several measures, including initial and sustained use of new technology by both counselors and patients. Additionally, this research indicates that seamlessly integrating organizational changes into existing workflows and using coaching to overcome hurdles and assess progress are important to improve implementation projects.

**Conclusions:**

At this interim point in the study, it appears that the use of the NIATx improvement process leads to better outcomes in implementation of changes within health care organizations. Moreover, some strategies used in this improvement process are particularly useful and should be drawn on more heavily in future implementation efforts.

**Trial Registration:**

ClinicalTrials.gov NCT03954184; https://clinicaltrials.gov/ct2/show/NCT03954184

## Introduction

This is a midterm report on a randomized trial comparing 2 different strategies for implementing innovations. Implementation and dissemination are ongoing challenges for innovations. While efforts to enhance theories in implementation science theory may help, we need to shift focus more to applying what we already know with efficiency and fidelity. We developed a simple and easily replicable implementation approach and are comparing it to a more traditional approach.

The challenges of implementation have been studied for hundreds (some would say thousands) of years by innovators such as Jethro of Midian [1], Heraclitus [2], Taylor [3], Box [4], Ishikawa [5], Batalden [6], Maslow [7], Phillips [8], Gantt [9], Deming [10], Delbecq [11], Mayo [12], Ohno [13], Maidque [14], Conner [15], Shewhart [16], Utterback [17], Fayol [18], Rogers [19], Van de Ven [20], Barnard [21], Barnett [22], Kanter [23], Gawande [24], Lewin [25], Cooper [26], Berwick [27], Argyris [28], Simon [29], Taguchi [30], Kotter [31], Hage [32], Kilbourne [33], Ackerman [34], and Damschroder [35].

A personal example may help elucidate why we cannot wait for more theory: Tim was 31 years old when he died alone in his room with a half-used syringe on his nightstand. He had fought opioid use disorder for almost half of his life. Ultimately, an act of kindness led to his demise. The last years of his life were among Tim’s best. He was in successful recovery. He had been clean and sober for nearly 5 years, reunited with his family, and gotten a job. He was 2 months away from getting his bachelor’s degree in brain and behavior studies and was planning to pursue a master’s degree. Both Tim’s dad (his favorite golfing partner) and his mom (the rock of the family) were hesitantly breathing a sigh of relief. Things were going well. But there was also a warning sign. Tim had stopped using Suboxone (a medication-assisted treatment designed to reduce the desire for opioids) because of side effects (terrible constipation and plummeting libido).

Then a friend of Tim’s called him from the hospital—her husband had survived an overdose and would soon be discharged. She knew he had heroin in their apartment. Would Tim search for it and clear the apartment before her husband’s return? Tim’s kindness drove him; he agreed to help. He sped to the hospital, got their key, returned to their apartment, and removed what he could find. Within hours of having heroin in his possession, for the first time in almost 5 years, Tim relapsed. He died from what was ultimately determined to be a “speedball” (a mixture of heroin and cocaine). Tim’s death devastated his family and friends. One of the authors of this paper attended the funeral. He sat next to a young person who was crying; we all were. The young person said, “Tim was my hope. If he couldn’t make it, how can I?” Tim had been trained in evidence-based cognitive behavioral therapy (CBT). One of CBT’s key principles relates to “seemingly irrelevant decisions” where one puts themselves in harm’s way without realizing it. Tim forgot that principle, and it killed him.

Computer-based technologies that could have helped Tim have been in place since the early 1980s. Examples include the Body Awareness Resources Network (BARN) [36], Computer Based Training for Cognitive Behavioral Therapy (CBT4CBT) [37], Treatment Evaluation Services (TES) [38], and Addiction Comprehensive Health Enhancement Support System (ACHESS) [39]. For instance, ACHESS contains brief reminders of CBT principles that might have called Tim’s seemingly irrelevant decision—to help his friend—to his attention. Alternatively, the weekly patient surveys in ACHESS might have called his attention to his increased anxiety and led him to think twice about his decision. However, few people with or without a substance use disorder [40] remember the CBT skills they have learned in treatment or have access to tools that could help them remember in the moments when those skills are most needed.

ACHESS, the original smartphone innovation that we used to compare implementation strategies, was designed to help counselors and patients. It offers a variety of services: communicate anonymously with peer support groups, help assess a patient’s relapse risk and link to interventions, use reminders to encourage adherence to therapeutic goals, privately communicate with the patient's counselor, provide addiction-related educational materials and tools, and send alerts if a patient visits a high-risk location such as a favorite bar.

**Figure 1:**
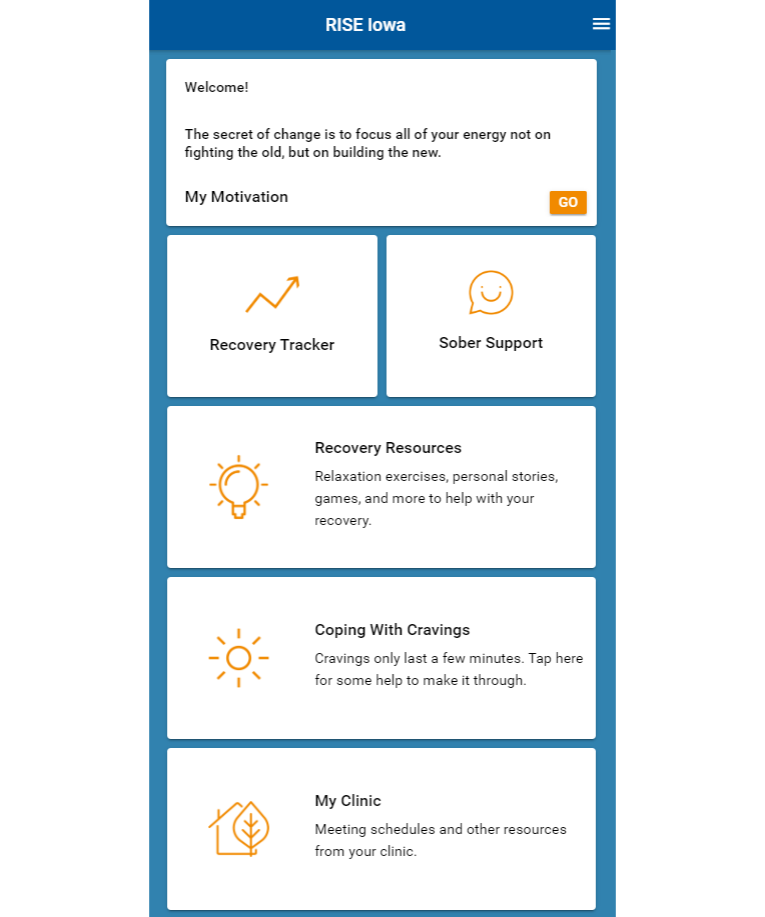
RISE Iowa (Recovering Iowans Supporting Each Other) app.

Several studies found that ACHESS reduced heavy drinking [41] and doubled retention in treatment [42]. However, only a few thousand of the millions of people facing substance use disorder (SUD) are using ACHESS or other technologically based systems. Hence, such a system provides an ideal target to compare implementation strategies. For this study, ACHESS was renamed RISE Iowa (Recovering Iowans Supporting Each Other) to make the app more appealing to treatment agencies in Iowa (Figure 1).

This paper seeks to answer the question, “What have we learned so far in this study about how to implement evidence-based practices?” Our ongoing randomized trial compares 2 strategies for widescale implementation, using RISE Iowa as the object of those implementation strategies.

## Methods

### Background

The first strategy is a typical product training approach where a sales representative introduces a product to key personnel in the adopting organization, trains key players on how to use the product, and offers a source for further support, such as training manual and computer accessible responses to frequently asked questions (FAQs). The second strategy adds a quality improvement (QI) methodology (the NIATx model [43]) that assigns an external coach who calls the agency once a month to monitor, support, and encourage the organization and uses a set of QI tools (eg, a checklist of steps to implement RISE Iowa at their site, flowchart to see how to integrate RISE Iowa into the organization’s workflow, and tools to predict and explain an organization’s readiness for change or to examine the potential of embedding the innovation). The NIATx model has been implemented by over 3500 addiction treatment organizations and was tested in a randomized trial involving over 200 addiction treatment agencies [44]. NIATx is designed to add structure and increase fidelity to the implementation process.

In the NIATx model, quality improvement coaches made 1 trip to each agency to get to know and train staff in RISE Iowa and to set implementation goals, followed by monthly Zoom calls to an organization’s change leader and change team. Coaches’ calls served as methods to assess progress and roadblocks, train and remind staff, celebrate successes, give feedback on progress, set follow-up goals, and identify and provide answers to questions and concerns. In addition, 4 times during the 18-month implementation period, organizations in the NIATx model were invited to convene via Zoom for more training and to share successes and challenges. Table 1 compares NIATx to the product training approach.

**Table 1:** Comparison of implementation strategies.

NIATx^a^	Product training
Coach and trainer conduct Zoom meeting with leadership of organization to review study and provide overview of RISE^b^ Iowa app.	Trainer conducts Zoom meeting with leadership of organization to review study and provide overview of RISE Iowa app.
Staff at organizational sites take survey regarding organization’s approach to change.	Staff at organizational sites take survey regarding organization’s approach to change.
Coach and trainer conduct Zoom meeting with change leader(s) and teams to review survey results and preview app.	N/A^c^
Trainer provides 2-hour staff training on the RISE Iowa app.	Trainer provides 2-hour staff training on the RISE Iowa app.
Coach provides 2-hour NIATx training.	N/A
Tech support is available via email.	Tech support is available via email.
Coach and trainer hold monthly coaching Zoom meeting with change leaders at study sites, in which coach shares ideas gained from working with other organizations.	N/A
Study staff emails staff and patients with RISE Iowa accounts regarding updates to the app.	Study staff emails staff and patients with RISE Iowa accounts regarding updates to the app.
Study staff send weekly and monthly emails providing data on new RISE Iowa accounts and RISE Iowa usage by staff and patients to change teams.	N/A
Additional resources are added to the app approximately monthly.	Additional resources are added to the app approximately monthly.
One additional cross-agency Zoom meeting is held with executive sponsors as well as 1 with change leaders. Additionally, 2 additional cross-agency training opportunities are offered	N/A

^a^NIATx: Network for the Improvement of Addiction Treatment.

^b^RISE: Recovering Iowans Supporting Each Other.

^c^N/A: Not applicable.

Coaches are individuals with experience in leading NIATx projects in their own organizations and who have received additional NIATx coaching training. Coaches have had at least 10 years of experience leading NIATx quality improvement projects. To assure fidelity and consistency among coaches, the coaches convened monthly with key study team members to review progress with the sites and discuss challenges and potential approaches to those challenges. Due to COVID-19 restrictions, coaches were unable to make in-person visits to 3 of the 11 agencies. Those agencies were trained via Zoom. A resource provided in the NIATx approach is a set of organizational surveys used to predict and explain the likelihood of successful RISE Iowa implementation [45] and assess chances that RISE Iowa will be sustained by the organization in the long run [46]. These analyses are designed to help coaches determine the strengths and challenges they will face in promoting adoption and sustainment at the agency and offer advice on how to overcome the challenges.

This paper presents interim results of our ongoing multicenter randomized trial in Iowa SUD treatment agencies but focuses mostly on lessons learned so far about implementation. No attempt was made to evaluate the RISE Iowa app itself. We are interested in implementation progress with NIATx compared to the product training approach. We expected the NIATx coaching calls alone would be superior, but we also wanted to explore what other aspects of the NIATx approach make a difference.

To compare the 2 implementation strategies, 11 Iowa-based addiction treatment organizations with 43 addiction treatment sites were randomly assigned to receive the product training or the NIATx approach. The sites’ progress is being tracked for an 18-month period during which aspects of the 2 implementation strategies are still active (eg, FAQs for product training, monthly coach calls for NIATx), followed by 10 months with no support to examine sustainability. The first cohort began the study intervention in 2019. The final cohort will complete the intervention by mid-2022.

Counselors and peer recovery coaches use RISE Iowa in a variety of ways, including by assigning material within it (eg, reading personal stories of others successfully struggling with SUD), monitoring patient progress by examining the weekly patient surveys, and participating in discussion groups. RISE Iowa automatically collects and stores data on how many patients sign up to use RISE Iowa and the amount of use by both counselors and patients. Counselors can track the data on patient use of RISE Iowa to better understand how well patients are following through on their recovery efforts.

In this report, we compare patient and counselor use of RISE Iowa between sites assigned to the NIATx approach versus product training only. RISE Iowa utilization is summarized as the average number of days logging into RISE Iowa per month and the percentage of patients or counselors who logged in per month.

To better understand the features of the NIATx approach that influenced implementation in this study, 3 coaches and 8 research staff conducted a combination of semistructured interviews and nominal group technique meetings [11,47] to identify and assess factors that played a role in the implementation and ones they would concentrate on if they were to implement RISE Iowa in another setting (or, in other words, what worked). These interviews identified 24 factors that were then evaluated by inviting coaches and research staff to select the 10 factors they considered to be most important in guiding adoption and sustainment. We prioritized these factors by counting the votes (from 11 possible voters) of each of the 24 factors received. We present the 10 factors that received the most votes alongside explanations provided by voters and summarize the number of votes received for the remaining 14 factors. We did not conduct a formal statistical analysis as this is preliminary data.

### Ethics Approval

This study received approval from Advarra Institutional Review Board (# 2018-0997). Interview participants provided oral informed consent.

## Results

### Use of RISE Iowa

Use of the RISE Iowa app was tracked over time for all participants. Here, we present the data collected so far for the first 5 months of each participants’ app usage after activating their account. The average number of days per months of app usage was calculated by tallying the number of days per month each participant opened the app and then calculating the mean of all tallies. The percent of active participants per month was calculated by dividing the number of participants who opened the app that month by the number of participants who had access to the app at that time point.

**Figure 2:**
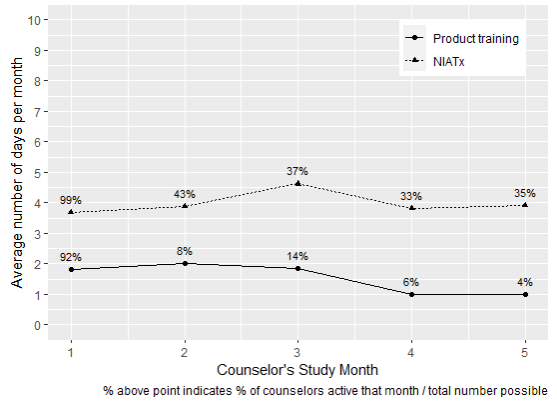
Counselors' average days of use of RISE Iowa (Recovering Iowans Supporting Each Other) app. NIATx: Network for the Improvement of Addiction Treatment.

Figure 2 compares counselor retention rates between the implementation approaches. By retention, we mean that counselors continue to log on to RISE Iowa after they are first introduced to it. For example, 24 of 68 (35%) of counselors and peer recovery coaches trained in RISE Iowa with the NIATx approach were still logging in 5 months later versus 2 of 51 (4%) who received product training. Furthermore, those 35% used RISE Iowa an average of about 4 days per week with NIATx versus an average of about 1.5 days per week in the product training arm. For the patient data, an account was removed from the analysis if it had been created on the day the analysis was run. For example, if person A created an account at 10 AM on day X and study staff downloaded the data at 4 PM on Day X, person A would have created an account the day that study staff downloaded the data, so person A would be removed from the analysis.

**Figure 3:**
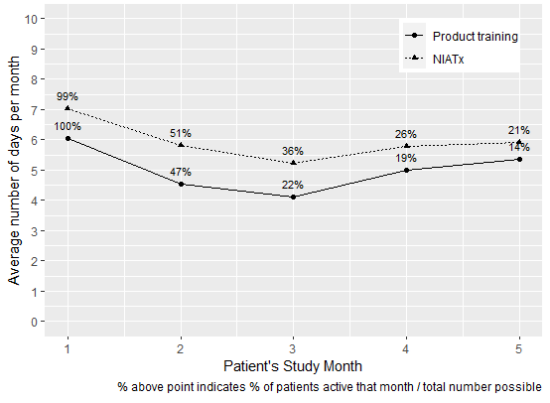
Patients' average days of use of RISE Iowa (Recovering Iowans Supporting Each Other) app. NIATx: Network for the Improvement of Addiction Treatment.

Figure 3 displays differences in patients’ use of RISE Iowa between the product training and NIATx approaches. The average days of RISE Iowa use per patient per month was about 6.5 at the NIATx sites versus about 5.5 in product training sites. Further, the number of patients using RISE Iowa is much smaller in the product training approach (11/81,14%) versus in the NIATx approach (150/722, 21%) at 5 months after each patient first logged on. Finally, it should be noted that the product training sites in this study treat 17% more patients than do the NIATx sites; however, there was a much smaller RISE Iowa enrollment in the product training locations. Table 2 shows the descriptive statistics for use of the RISE Iowa app by participants.

**Table 2:** Descriptive statistics for use of the RISE Iowa app by participants.

Type of RISE^a^ Iowa user	Arm	Study month	Number of days per month, mean (SD)	People, n	People possible, n	People active, %
Patients	Product training	1	6.05 (5.53)	82	82	100
Patients	Product training	2	4.53 (5.31)	38	81	47
Patients	Product training	3	4.11 (3.61)	18	81	22
Patients	Product training	4	5 (8.27)	15	81	19
Patients	Product training	5	5.36 (8.58)	11	81	14
Patients	NIATx^b^	1	7.02 (6.25)	792	797	99
Patients	NIATx	2	5.83 (6.74)	402	789	51
Patients	NIATx	3	5.24 (6.34)	276	763	36
Patients	NIATx	4	5.79 (7.55)	193	749	26
Patients	NIATx	5	5.92 (7.54)	150	722	21
Counselors	Product training	1	1.81 (1.45)	47	51	92
Counselors	Product training	2	2 (1.41)	4	51	8
Counselors	Product training	3	1.86 (1.46)	7	51	14
Counselors	Product training	4	1 (0)	3	51	6
Counselors	Product training	5	1 (0)	2	51	4
Counselors	NIATx	1	3.67 (2.94)	69	70	99
Counselors	NIATx	2	3.87 (3.42)	30	70	43
Counselors	NIATx	3	4.62 (5.28)	26	70	37
Counselors	NIATx	4	3.83 (5.77)	23	70	33
Counselors	NIATx	5	3.92 (4.80)	24	68	35

^a^RISE: Recovering Iowans Supporting Each Other.

^b^NIATx: Network for the Improvement of Addiction Treatment.

### Factors Influencing Implementation

We explored what it is about the NIATx approach that is thus far leading to better results. Ordered by total number of votes received, the top factors are (including the number of votes each received) are as follows.

First, identify patient and counselor pain points and show how RISE Iowa helps to relieve that pain: 10 votes out of a possible 11. This factor creates a value proposition on why the change should be adopted. As a counselor put it, “From the beginning, I’ve really tried to make [RISE Iowa] something that will feel useful. I am trying to have our IT [person] put as many relevant things on RISE Iowa as possible [to keep it fresh].” Counselor pain points may include busyness, demands of their job, and having to accomplish several required activities with each patient. Patient pain points include factors that inhibit recovery, such as cravings, triggers, and isolation.

Second, find ways to integrate RISE Iowa into the organization’s standard workflow: 9 votes. Another counselor said, “I've been more intentional about going through my case load prior to the monthly coaching calls asking: ‘Who are the eligible folks and why aren't they signed up with RISE Iowa yet?’. That's a trigger for me to say, ‘Here are the folks that I need to target.’“ As such, the counselor has made RISE Iowa enrollments part of their standard workflow.

Third, use coaches to motivate, help overcome hurdles, assess progress, and share learnings across the sites: 8 votes. A coach said, “Clinicians are asked to manage many competing demands as they assist patients in their recovery. Adding a tool [like RISE Iowa] to their workflow takes mental energy and focus. Coaches trained agency staff in NIATx process improvement and the associated tools. With coaches available, this means that providers do not need to have all the answers as they pilot test improvements in their workflows.”

Fourth, get senior executives to encourage agency staff to try RISE Iowa: 8 votes. Another coach said, “Leaders can encourage staff to make RISE Iowa part of their regular client intake and treatment process. Leaders can also free staff time to use RISE Iowa themselves and become familiar with its features.”

Fifth, get bugs out of the RISE Iowa app and make it user friendly: 7 votes. A researcher said. “I just know that nothing is more of a deterrent than trying to use something that doesn’t work.”

Sixth, provide data and administrative support: 6 votes. A coach said, “Administrative staff offer real-time technical support and training to agencies, as well as data on RISE Iowa adoption and use. It was easier for organizations to justify the allocation of limited resources to support sustainment after staff provided data demonstrating measurable progress.”

Seventh, use testimonials to reinforce the value of RISE Iowa: 5 votes. A coach said, “In my 20 years of facilitating organizational change, stories/testimonials are among the most impactful resources. To hear a story from someone you identify with can provide a path for change and a belief that a change might be worth the effort.”

Eighth, have cross-agency calls to share learnings and concerns: 5 votes. A research staff member noted that “cross agency calls provide a way for counselors and agency staff to learn from each other. Nothing works better than to hear good things from a colleague.”

Ninth, use financial incentives: 5 votes. A researcher noted that “incentives (such as lotteries) can motivate people toward chosen behaviors. Incentives can tip the scales away from competing priorities.”

Tenth, remind and retrain both counselors and patients to use RISE Iowa: 5 votes. One counselor said, “The 'coping with cravings’ stuff; when I first heard about that, I thought, ‘this is beautiful!’ But that had not been [an] area that I really tapped into. Its presence makes me think of a lot of new things that I could be doing with RISE Iowa.”

Other factors and the number of votes they received (indicated in parenthesis) are listed as follows:

1. Assess organizational readiness for change to learn what areas they need to work on to have the best chance of successful implementation (4).
2. Get many patients on RISE Iowa right away to reach critical mass for discussion groups because there needs to be enough people to have an active discussion (4).
3. Find ways to address the digital divide (4).
4. Give agencies tools they can use to improve quality (4).
5. Protect privacy (3).
6. Celebrate successes (2).
7. Make access to RISE Iowa a privilege. Making people invest to participate makes a system more appealing (1).
8. Do not prejudge who will use RISE Iowa; clinicians may pick and choose which patients are best suited for a recovery app and may misjudge (1).
9. Use lessons from the COVID-19 pandemic. For instance, virtual visits were found to be effective (1).
10. Coaches should make 1 in-person visit if possible (1).
11. Refresh RISE Iowa often to keep it interesting and up to date (1).
12. Make RISE Iowa free of charge (1).
13. Ignore skeptics; however, ignoring skeptics would probably be a mistake because they likely have important insights to offer (0).
14. Do not use inflammatory words, such as “track.” The “recovery tracker” on the app could be seen as an inflammatory term because people do not like to feel like they are being watched (0).

## Discussion

### Interim Findings

This study’s interim results suggest that the NIATx process is especially useful for integrating RISE Iowa within existing workflows and helping clinicians address the challenges they already face at work. It is further notable that in an earlier randomized trial [48] involving 201 addiction treatment agencies across 4 states, we explored whether processes improved more in agencies that received the product training approach versus a full improvement collaborative (ie, periodic face-to-face meetings, coaches, joint calls with all agencies at once) versus joint improvement calls only versus monthly coaching calls with individual agencies, with the latter method used in this study. We found that monthly coaching calls (the NIATx approach) worked at least as well as the full collaborative and better on both cost and effectiveness outcomes than the other arms.

The results of this study point to the importance of identifying and responding to the pain points faced by the implementing organization, along with finding a way to integrate the new technology easily into the organization’s workflow. The more that staff are asked to change their workflow, the less likely the implementation will be to succeed.

Other research finds that the average commercial app loses 77% of its users within the first 3 days after installation. Within 30 days, it has lost 90% of users. Within 90 days, it has lost over 99% [49]. In contrast, in earlier tests of ACHESS in Federally Qualified Health Centers, over 60% of actively enrolled people continued using ACHESS 4 months after enrolling [44].

Therefore, we were surprised at this study’s retention rate of only 22% at 5 months. Multiple reasons may have influenced this higher-than-expected dropout rate. The first is that in prior studies, we typically delayed offering ACHESS (system on which RISE Iowa is based) until the patient demonstrated their commitment to recovery by attending at least 3 clinic visits. In this study, many clinics offered RISE Iowa at the first visit. As it turns out, the dropout rate from treatment after the first visit is high, typically about 30% to 50% in the first month [50,51]. Hence, by the time we offered ACHESS in prior studies, only 50% to 70% of the initial patients remained in treatment, and the retention rate among that 50% to 70% of the original population was closer to 28% to 38%. Second, 25% of Iowa residents live in rural areas with no or inadequate access to the internet [52], moving retention rates of potential patients closer to 50% [53]. This study was also conducted during the COVID-19 pandemic, which could have had varied and unpredictable influences on patient retention and on counselors’ ability to introduce patients to the app. Considering these factors, a lower retention rate in this interim analysis is not surprising compared to previous studies of ACHESS.

As the voting results demonstrate, we value the use of financial incentives [54] to implement and sustain innovations. However, our counselors and clinic staff felt that incentives would not be their first choice, if given a choice. When we raised with agencies the option to incentivize counselors and patients with our own grant money, they rejected the option in most cases. Moreover, substantial literature supports the importance of deeply understanding our customers (patients, agencies, and staff) above all [55]. When designing RISE Iowa, we employed critical incident interviews [56] and conducted walk-throughs [57] to understand what it is like to be a patient, a counselor, and an agency administrator. This effort helped us create a value proposition that makes it in the agency’s best interest to adopt and use the technology. These efforts resulted in our team simplifying RISE Iowa adoption and ways to use it as much as possible and understanding where RISE Iowa fits in the workflow to make it as easy to adopt as possible. As one counselor said, “I am so busy that if I have to so much as lift a finger to use RISE Iowa, I won’t do it.”

NIATX coaches provided strategic support to the agencies’ implementation efforts. During their monthly phone sessions, coaches attempted to build motivation to use RISE Iowa by stressing how RISE Iowa can help address “pain” points. Coaches encouraged executives to support RISE Iowa, addressed resistance, and removed barriers while stressing the need to build care delivery systems that integrate RISE Iowa into the clinical and administrative workflows. Coaches also made their organizations aware of evidence-based practices, such as contingency management and motivational interviewing, which might facilitate RISE Iowa use. Overall, with just 1 phone call per month, coaches helped organizations over the rough patches and provided persistent motivational support to expand RISE Iowa use.

### Limitations

This report has several limitations. First, these are preliminary findings during the intervention phase of the trial. Therefore, our results do not include the sustainability of postintervention RISE Iowa utilization. However, the primary purpose of the paper is to delve more deeply into reasons why a process like NIATx would be superior, not a definitive response on the superiority of the intervention. Second, the qualitative results do not include patient perspectives. This study focused on organization implementation [58] aspects of RISE Iowa app use. Accordingly, these results are from clinician, organizational leader, and coach perspectives. Future studies should also include patient perspectives. The people we did include bring an important perspective that needs to be understood. Third, this study was not designed to address the effectiveness of the RISE Iowa app. Past research addressed this issue [44,59]. Fourth, while there were 43 different sites in this study, there were only 11 different organizations. While most of the sites operated independently of their senior leadership, there were times when corporate policy limited independence. In that sense, the number of truly independent units is smaller than might initially appear. Finally, there was a site dropout rate of 7%, and the study design had a planned dropout rate of 20%. We find this to be a reasonable rate due to the impact that personnel changes may had have on needed participation in the research trial. In addition, the organization that dropped out was replaced with an organization with similar characteristics used for randomization.

### Conclusions

As mentioned earlier, this interim report describes what we have found so far. The results imply that the NIATx approach, along with the use of coaching, led to higher adoption by both patients and counselors, but we are awaiting final results, which are approximately 6 months away. After receiving our final results, we will seek to understand more than we do today, particularly about how well RISE Iowa was sustained in this project.

We hope readers will find this interim report valuable. Finally, we want to reinforce our belief that finding easy-to-use tools to reliably implement evidence-based practice is more important today than adding to theory. People need help now.

## Appendix

Multimedia Appendix 1 CONSORT-eHEALTH checklist (V 1.6.1).

## Abbreviations

ACHESS: Addiction Comprehensive Health Enhancement Support System
BARN: Body Awareness Resources Network
CBT: cognitive behavioral therapy
CBT4CBT: Computer Based Training for Cognitive Behavioral Therapy
FAQ: frequently asked question
NIATx: Network for the Improvement of Addiction Treatment
NIDA: National Institute on Drug Abuse
NIH: National Institutes of Health
QI: quality improvement
RISE Iowa: Recovering Iowans Supporting Each Other
SUD: substance use disorder
TES: Treatment Evaluation Services

## Data Availability

The data sets generated and analyzed in this study are not publicly available due to the complexity of app use data and personal information embedded in the data set, but they are available from the corresponding author on reasonable request.
